# Psychometric properties and measurement invariance of the Turkish version of the multidimensional cognitive attentional syndrome scale

**DOI:** 10.3389/fpsyg.2026.1787616

**Published:** 2026-05-15

**Authors:** Kadir Ozdel, Sukru Erkil Cetinel, Sedat Batmaz, Ali Ercan Altinoz

**Affiliations:** 1 Department of Psychiatry, Ankara Etlik City Hospital, University of Health Sciences (Saglik Bilimleri Üniversitesi), Ankara, Türkiye; 2 Department of Psychiatry, Ankara Etlik City Hospital, Ankara, Türkiye; 3 Department of Psychology, Ankara Social Sciences University, Ankara, Türkiye; 4 Department of Psychiatry, Eskisehir Osmangazi University, Eskisehir, Türkiye

**Keywords:** cognitive, cognitive attentional syndrome, measurement invariance, metacognitive, psychotherapy

## Abstract

**Background:**

Cognitive attentional syndrome and metacognitive beliefs are the two elements that comprise the metacognitive therapy model. The aim of the Multidimensional Cognitive Attentional Syndrome Scale (MCASS) is to comprehensively measure this construct.

**Aim:**

To test the validity and reliability of the Turkish version of the MCASS and its use in patients and controls.

**Methods:**

A total of 229 participants (115 patients and 114 healthy controls) were included in this study. Confirmatory factor analyses, internal consistency analyses (Cronbach’s *α* and McDonald’s *ω*), concurrent validity analyses (correlations with relevant measures), test–retest analyses, and measurement invariance analyses were conducted to determine the validity and reliability of the Turkish MCASS.

**Results:**

Confirmatory factor analyses supported the seven-factor structure of the MCASS after excluding item 7 in the patients and controls. The internal consistency was acceptable to excellent (Cronbach’s *α* = 0.694–0.952 in patients, 0.695–0.954 in controls; McDonald’s *ω* = 0.695–0.954 and 0.737–0.948, respectively). Test–retest reliability was moderate to good (ICC = 0.620–0.741 for subscales; ICC = 0.705 for total score). The correlations between the MCASS subscales and the related constructs were generally moderate (*r* = 0.316–0.522) except for external fixation. In terms of measurement invariance across clinical and non-clinical groups. Results supported configural and metric invariance, while partial support was found for strict invariance.

**Conclusion:**

The Turkish modified 20-item MCASS is a suitable tool for clinical and non-clinical settings. However, diagnosis-specific cognitive attentional syndrome scales are needed for comprehensive assessment.

## Introduction

Metacognitive therapy (MCT) targets dysfunctional metacognitive beliefs (MCB) and self-regulatory processes involved in executive function (Wells and Matthews, 1996). The role of metacognition in the maintenance of emotional disorders is central. Cognitive attentional syndrome (CAS) maintains problematic metacognitions. Unlike conventional cognitive therapy, the CAS components outlined by Wells (2011), including repetitive thinking (worry and rumination), threat monitoring, and maladaptive behavioral strategies, are considered metacognitive strategies. These responses, including physical (behavioral) avoidance, are related to metacognition. However, repetitive self-referential thinking (i.e., worry and rumination) plays a central role in the maintenance of emotional disorders because it uses the cognitive, attentional, and behavioral resources needed to evaluate dysfunctional beliefs and emotional processing (Sibrava and Borkovec, 2006; Mathews, 1990). Thus, these two constructs, namely MCB and the CAS, are the primary intervention areas (Batmaz et al., 2021) in MCT, which is an effective treatment option for various psychological disorders (Normann and Morina, 2018; Philipp et al., 2019).

Nordahl and Wells (2019) first developed the CAS-1 scale to measure the components of the CAS. This scale was translated into a Turkish version that was investigated by Gündüz et al. (2019). The CAS-1 scale was proposed as a one-factor tool; however, specific items to measure the CAS components have been reported (Fergus et al., 2012). The CAS-1 scale does not comprehensively address maladaptive strategies. For example, there are many statements that include vague phrases such as “avoided situations,” “asked for reassurance,” “tried not to think about things,” and “tried to control my emotions.” It also attempts to measure MCBs (e.g., “worrying helps me cope,” “focusing on possible threat can keep me safe,” and “it is important to control my thoughts”). To address these shortcomings, the Multidimensional Cognitive Attentional Syndrome Scale (MCASS) was developed by Conboy et al. (2021) allowing for comprehensive measurements of the CAS components. The MCASS measures CAS components under seven subjects including worry, rumination, internal threat monitoring, external threat monitoring, thought suppression, physical avoidance behavior, and substance use.

Investigating theoretical backgrounds of a psychotherapy model in various cultures requires high-quality measurement tools to measure the main elements of that model in each culture. The measurement tool must also work well in individuals with any mental disorder as well as healthy controls. Currently, the psychometric properties of the MCASS have been investigated in its original language, and an Arabic adaptation has been created (Helmy et al., 2023). This study aimed to adapt the MCASS to the Turkish culture and to examine its psychometric properties. We tested the fit of the seven-factor MCASS data from both patients and controls in a construct validity test. We specifically investigated internal consistency, positive inter-correlations, convergent validity, and stability over time.

## Materials and methods

### Patient selection

The local ethical committee approved this study (31.01.2023/AESH-EK1-2023-731). Written and verbal informed consent were obtained from the included participants.

### Inclusion criteria

Patients who presented to the Ankara Etlik City Hospital Psychiatry Outpatient Clinic were offered study inclusion if they were between 18 and 65 years of age and able to provide informed consent for voluntary participation in the study. For inclusion in the clinical group, the patients were required to have any mental health complaint. For inclusion in the control group, the patients were required to have no current psychiatric disorder. The two groups were age- and sex-matched. Sample size adequacy was evaluated based on recommendations suggesting a minimum of five participants per item for factor analytic studies. Given that the scale consists of 21 items, the total sample of 229 participants (approximately 11 participants per item) exceeded this criterion, indicating that the sample size was sufficient for the planned analyses.

The clinical sample comprised 38 individuals with a primary diagnosis of panic disorder, 41 with generalized anxiety disorder, 31 with major depressive disorder, 3 with social anxiety disorder, and 2 with obsessive-compulsive disorder. Clinical diagnoses were made by experienced clinicians (M.D.s) in accordance with DSM-5 criteria.

The control group included individuals who did not report any psychiatric complaints. The absence of psychiatric conditions was determined through review of hospital records and participants’ declarations.

Since symptom severity was not assessed with standardized instruments, the findings should be interpreted with caution in this respect.

### Exclusion criteria

Exclusion criteria were substance use disorder, intellectual disability, less than 11 years of formal education (to ensure a minimum intellectual/educational level for valid scale completion), psychotic disorder, or bipolar disorder (manic, depressive, mixed, or hypomanic episode).

### Measuring tools

#### Sociodemographic data form

This form was developed by the authors to determine the participants’ basic sociodemographic data and capture all relevant personal and clinical information.

#### MCASS

The MCASS is a seven-factor, Likert-type self-report scale that was developed by Conboy et al. (2021). The seven subscale factors are: (1) Worry; (2) Rumination; (3) Internal threat monitoring; (4) External threat monitoring; (5) Thought suppression; (6) Physical avoidance behavior; and (7) Substance use.

#### Multidimensional experiential avoidance questionnaire

The original 62-item Multidimensional Experiential Avoidance Questionnaire (MEAQ) scale was developed by Gámez et al. (2011) and was later shortened to a 30-item form (MEAQ-30) by Sahdra et al. (2016). The Turkish MEAQ-30 was tested for validity and reliability by Ekşi et al. (2018). The scale is based on contextual behaviorism and the acceptance commitment therapy model (Hayes et al., 2012). It measures experiential avoidance, which is one of the six elements of psychological inflexibility that also includes behavioral avoidance, distress aversion, procrastination, distraction and suppression, repression/denial, and distress endurance.

#### Depression anxiety stress scale

Lovibond (1983) developed the Depression Anxiety Stress Scales (DASS-21) to measure the most common symptoms of mental disorders (i.e., depression, anxiety, and distress). The Turkish DASS-21 was tested for validity and reliability by Sariçam (2018).

#### Ruminative response scale

The Ruminative Response Scale (RRS-10) was developed by Nolen-Hoeksema and Morrow (1991) and is based on response style theory. It measures the ruminative thinking style, which is the most associated with psychopathology among response styles. The scale has two subscales, namely brooding and reflection, with a Likert-type scale. The Turkish RRS-10 was tested for validity and reliability by Erdur-Baker and Bugay (2010).

#### Metacognitive questionnaire

Wells and Cartwright-Hatton (2004) developed the metacognitive questionnaire (MCQ-30). It is based on the metacognitive model (Wells, 2011) to measure metacognition in five areas including positive beliefs about worry, negative beliefs about the uncontrollability and dangerousness of thoughts, cognitive (attention and memory) confidence, the need to control thoughts, and cognitive self-consciousness. Tosun and Irak (2008) conducted validity and reliability studies on the Turkish MCQ-30.

#### CAS-1

The scale CAS-1 consists of 16 items and one or two factors. It was developed by Wells and Cartwright-Hatton (2004) and is based on the metacognitive model (Wells and Cartwright-Hatton, 2004). The first item measures the level of repetitive thinking (i.e., worry and rumination). The second item measures threat monitoring. Items 3–8 measure six specific CAS strategies with the following sentences: (1) I avoided situations; (2) I asked for reassurance; (3) I tried not to think about things; (4) I tried to control my emotions; (5) I used alcohol/drugs; and (6) I controlled my symptoms. These are all measurements of backfiring coping strategies. Items 9–16 measure MCB including uncontrollability, danger, and positive beliefs. Overall, the CAS-1 measures CAS components as well as dysfunctional MCBs. The Turkish CAS-1 was tested for validity and reliability by Gündüz et al. (2019).

#### Translation and adaptation

Permission to use and translate the MCASS into Turkish was obtained from Joseph R. Bardeen, one of the developers of the scale and the corresponding author of the original study.

The translation and cultural adaptation process followed a multi-step procedure. Initially, three independent forward translations were obtained from MCT therapists who were fluent in both English and Turkish. These translations were reviewed and synthesized into a single Turkish version by a bilingual expert with domain knowledge in MCT.

The reconciled Turkish version was then back-translated into English by an independent bilingual expert who was blinded to the original instrument. The back-translated version was compared with the original scale to ensure semantic and conceptual equivalence.

Subsequently, both the Turkish translation and the original version were sent to three independent experts in MCT to be evaluated in terms of clarity and theoretical consistency. Based on their feedback, the authors re-evaluated the wording and finalized the Turkish version for use in this study.

In addition, a pilot evaluation was conducted to assess item clarity and comprehensibility.

### Data analysis

Descriptive statistics (frequency and percentage, mean and standard deviation) were calculated to describe the patient group. Confirmatory factor analysis (CFA), convergent validity, and discriminant validity analyses were performed to provide evidence for the validity of the measurements obtained from the Turkish MCASS. Multigroup CFA analysis was performed to test the representation of the patient and control groups. The *χ*^2^ test was used for categorical variables, and the independent samples *t* test was used for continuous variables in the comparison of the groups. Cronbach’s alpha and McDonald’s omega internal consistency coefficients were used to provide evidence for the reliability of the measurements obtained from the MCASS. Finally, correlation analyses between the theoretically related measures were performed to determine the convergent validity. No formal correction for multiple testing (e.g., Bonferroni) was applied, as the primary focus was on the pattern and magnitude of the correlations rather than isolated significance tests.

Before proceeding to CFA some assumptions were tested for consistent and accurate results of the obtained estimates. First, missing data analysis was performed on the data set. Missing data was present for some items, but it did not exceed 5%. The expectation maximization algorithm was used to assign values because it provides effective parameter estimation and more unbiased results when the amount of missing data is small (Enders, 2013). Even though extreme values might affect the results, none were present.

The multivariable skewness (Zç) and kurtosis (Zb) values, the *χ*^2^ value for multivariable skewness and kurtosis, and the relative multivariate kurtosis values were calculated to test the multivariable normal distribution assumption of the data set. Multivariate normality was not present. Therefore, the robust maximum likelihood method was used in CFA. CFA analyses were performed using the LISREL (version 8.8) statistical package program.

Pairwise correlations were examined, and there were no correlations greater than 0.80, indicating no multicollinearity problem and a linear relationship between variables. The current sample size met the threshold of at least five participants per item. The test–retest reliability analyses were conducted in a group of controls (*n* = 23) after 2 weeks since the MCASS scores could change over time.

## Results

### Demographics

A total of 229 participants were recruited for this study. The patient group included 115 patients with a mean age of 25.36 ± 3.79 years and was 72.8% female.

The control group included 114 healthy controls with a mean age of 25.73 ± 8.48 years and was 81.7% female (Supplementary Table S1). Participants for the test–retest reliability analysis were selected through convenience sampling of the control group.

### Validity analyses

#### Patient group

The CFA analysis was conducted for the Turkish culture suitability of the measurement model defined for the MCASS for both the control and patient groups. The model data fit values obtained for the analysis of the patient group are presented in Table 1. The other fit indices were acceptable except for the Goodness of Fit Index (GFI) and Normed Fit Index (NFI) values. The low GFI and NFI values were due to the data set not meeting the multivariate normality condition. The Comparative Fit Index (CFI) and Non-Normed Fit Index (NNFI) indices were used instead of GFI and NFI, respectively (Cheung and Rensvold, 2002).

**Table 1:** Estimated values for the multidimensional cognitive attentional syndrome scale and threshold values of fit indices.

Fit index	Good fit	Acceptable fit	Estimations
S–B *χ*^2^ df	0 ≤ *χ*^2^/df < 2	2 ≤ *χ*^2^/df ≤ 5	231.518/149 = 1.554
RMSEA	0 ≤ RMSEA < 0.05	0.05 ≤ RMSEA ≤ 0.10	0.069
NNFI	0.95 ≤ NNFI ≤ 1.00	0.90 ≤ NNFI < 0.95	0.937
NFI	0.95 ≤ NFI ≤ 1.00	0.90 ≤ NFI < 0.95	0.876
CFI	0.95 ≤ CFI ≤ 1.00	0.90 ≤ CFI < 0.95	0.951
GFI	0.95 ≤ GFI ≤ 1.00	0.90 ≤ GFI < 0.95	0.836
SRMR	0 ≤ SRMR < 0.05	0.05 ≤ SRMR ≤ 0.10	0.062

CFI, The Comparative Fit Index; df, Degrees of freedom; GFI, Goodness of fit index; NFI, Normed fit index; NNFI, Non-normed fit index; RMSEA, Root mean square error of approximation; S–B, Satorra–Bentler chi-square; SRMR, Standardized root mean square residual.

The estimates of the path coefficients for the tested model revealed that the factor loading of item seven was not statistically significant (*p* = 0.398). Hence, it was removed from the measurement tool, and the analysis was repeated (Table 2). The factor loadings (*λ* = 0.503–0.978) and error variance values (*ε* = 0.010–0.747) of the items in the measurement tool had acceptable values. The factor loadings of the items of 0.30 and above indicated that they were suitable items for measuring the latent structure, and the error variances less than 0.90 indicated an acceptable error in estimating the latent structure (Kline, 2023).

**Table 2:** Factor loading values for the patient group.

Factor	Indicator	Estimate	Standard error	*Z* value	*p* value	95%CI		Standard estimate (all)
						Lower	Upper	
1	MCASS1	1.112	0.105	10.545	0.001	0.905	1.319	0.827
	MCASS2	1.413	0.120	11.774	0.001	1.178	1.648	0.889
	MCASS3	1.389	0.125	11.104	0.001	1.144	1.634	0.856
2	MCASS4	1.402	0.128	10.932	0.001	1.151	1.653	0.829
	MCASS5	1.667	0.115	14.444	0.001	1.441	1.893	0.978
	MCASS6	1.641	0.110	14.929	0.001	1.426	1.856	0.995
3	MCASS8	1.199	0.158	7.567	0.001	0.888	1.509	0.765
	MCASS9	1.136	0.163	6.973	0.001	0.817	1.455	0.696
	MCASS10	1.238	0.116	10.686	0.001	1.011	1.465	0.824
4	MCASS11	1.437	0.112	12.796	0.001	1.217	1.657	0.923
	MCASS12	1.564	0.115	13.622	0.001	1.339	1.789	0.957
5	MCASS13	1.194	0.128	9.339	0.001	0.943	1.445	0.783
	MCASS14	1.349	0.124	10.837	0.001	1.105	1.593	0.874
	MCASS15	1.065	0.142	7.493	0.001	0.787	1.344	0.661
6	MCASS16	0.778	0.142	5.490	0.001	0.500	1.055	0.503
	MCASS17	1.343	0.113	11.920	0.001	1.122	1.564	0.928
	MCASS18	1.226	0.121	10.095	0.001	0.988	1.464	0.823
7	MCASS19	1.771	0.148	11.995	0.001	1.482	2.060	0.913
	MCASS20	1.390	0.156	8.930	0.001	1.085	1.695	0.742
	MCASS21	1.560	0.147	10.623	0.001	1.273	1.848	0.841

CI, Confidence interval; MCASS, Multidimensional Cognitive Attentional Syndrome Scale.

#### Control group

Analyses were performed for the control group, and the fit values are presented in Table 3. All fit indices were acceptable except for the GFI and NFI values. The low GFI and NFI values were due to the data set not meeting the multivariate normality condition. The CFI and NNFI indices were used instead of GFI and NFI (Cheung and Rensvold, 2002).

**Table 3:** Estimated values for the multidimensional cognitive attentional syndrome scale and cutoff values of fit indices for the control group.

Fit indexes	Good fit	Acceptable fit	Estimations
S–B *χ*^2^/df	0 ≤ *χ*^2^/df < 2	2 ≤ *χ*^2^/df ≤ 5	239.188/149 = 1.605
RMSEA	0 ≤ RMSEA < 0.05	0.05 ≤ RMSEA ≤ 0.10	0.073
NNFI	0.95 ≤ NNFI ≤ 1.00	0.90 ≤ NNFI < 0.95	0.920
NFI	0.95 ≤ NFI ≤ 1.00	0.90 ≤ NFI < 0.95	0.853
CFI	0.95 ≤ CFI ≤ 1.00	0.90 ≤ CFI < 0.95	0.937
GFI	0.95 ≤ GFI ≤ 1.00	0.90 ≤ GFI < 0.95	0.836
SRMR	0 ≤ SRMR < 0.05	0.05 ≤ SRMR ≤ 0.10	0.074

CFI, The Comparative Fit Index; df, Degrees of freedom; GFI, Goodness of fit Index; NFI, Normed Fit Index; NNFI, Non-Normed Fit Index; RMSEA, Root Mean Square Error of Approximation; S–B, Satorra–Bentler chi-square; SRMR, Standardized Root Mean Square Residual.

The estimates of the path coefficients for the tested model revealed that the factor loading of item seven was not statistically significant. Therefore, it was removed from the measurement tool, and the analysis was repeated (Table 4). The factor loadings (*λ* = 0.450–0.944) and error variance values (*ε* = 0.010–0.747) of the items in the measurement tool had acceptable values. The factor loadings of the items at 0.30 and above indicated that they were suitable items for measuring the latent structure, and the error variances less than 0.90 indicated an acceptable amount of error in measuring the latent structure (Kline, 2023).

**Table 4:** Factor loading values for the control group.

Factor	Indicator	Estimate	Standard error	*Z* value	*p* value	95%CI		Standard estimate (all)
						Lower	Upper	
1	MCASS1	1.063	0.117	9.069	<0.001	0.833	1.293	0.757
	MCASS2	1.293	0.118	10.985	<0.001	1.062	1.523	0.866
	MCASS3	1.298	0.128	10.124	<0.001	1.047	1.549	0.819
2	MCASS4	1.092	0.091	12.034	<0.001	0.914	1.270	0.888
	MCASS5	1.205	0.090	13.336	<0.001	1.028	1.383	0.944
	MCASS6	1.104	0.083	13.354	<0.001	0.942	1.266	0.945
3	MCASS8	0.924	0.136	6.792	<0.001	0.657	1.191	0.757
	MCASS9	1.055	0.153	6.874	<0.001	0.754	1.355	0.770
4	MCASS10	0.958	0.112	8.557	<0.001	0.739	1.178	0.717
	MCASS11	1.186	0.101	11.799	<0.001	0.989	1.383	0.894
	MCASS12	1.195	0.100	11.952	<0.001	0.999	1.390	0.902
5	MCASS13	1.182	0.109	10.826	<0.001	0.968	1.396	0.859
	MCASS14	1.193	0.114	10.462	<0.001	0.969	1.416	0.839
	MCASS15	1.007	0.116	8.666	<0.001	0.780	1.235	0.733
6	MCASS16	0.684	0.142	4.815	<0.001	0.405	0.962	0.450
	MCASS17	1.341	0.116	11.565	<0.001	1.114	1.568	0.943
	MCASS18	1.080	0.127	8.516	<0.001	0.832	1.329	0.739
7	MCASS19	1.210	0.110	10.967	<0.001	0.994	1.426	0.851
	MCASS20	1.160	0.108	10.706	<0.001	0.948	1.373	0.838
	MCASS21	1.338	0.111	12.006	<0.001	1.120	1.557	0.903

CI, Confidence interval; MCASS, Multidimensional Cognitive Attentional Syndrome Scale.

Item 7 was excluded from the Turkish form due to both conceptual and psychometric considerations. In the non-clinical group, Item 7 showed a very low standardized factor loading (*λ* = 0.194, *p* = 0.069), and its 95% confidence interval included zero. Similarly, in the clinical group, Item 7 demonstrated a negligible standardized loading (*λ* = 0.090, *p* = 0.398), with a 95% confidence interval also including zero. In contrast, the remaining items loaded significantly and substantially on their intended factors. These findings indicated that Item 7 did not function adequately in either group.

### Reliability analyses

Internal consistency reliability was performed. Cronbach’s alpha and McDonald’s omega coefficients were calculated for the internal consistency reliability of the MCASS (Table 5).

**Table 5:** Estimated reliability values of the multidimensional cognitive attentional syndrome scale.

Subscales	Patient		Control	
	Cronbach’s *α*	McDonald’s *ω*	Cronbach’s *α*	McDonald’s *ω*
MCASS-Rumination (F1)	0.889	0.896	0.855	0.856
MCASS-Substance use (F2)	0.952	0.954	0.946	0.948
MCASS-External fixation (F3)	0.694	0.695	0.733	0.737
MCASS-Thought suppression (F4)	0.928	0.933	0.872	0.880
MCASS-Behavioral avoidance (F5)	0.819	0.809	0.849	0.854
MCASS-Internal fixation (F6)	0.764	0.820	0.713	0.783
MCASS-Worry (F7)	0.869	0.875	0.896	0.902

F1, Factor 1; F2, Factor 2; F3, Factor 3; F4, Factor 4; F5, Factor 5; F6, Factor 6; F7, Factor 7. MCASS, Multidimensional Cognitive Attentional Syndrome Scale.

### Measurement invariance

After providing evidence for the construct validity of the measurement tool for both groups, measurement invariance was assessed across clinical and control groups using multiple-group CFA. This approach follows a hierarchical procedure in which each level of invariance must be established before proceeding to the next.

Measurement invariance was tested progressively. First, configural invariance was established, confirming that the multi-factorial structure was the same across groups. The analyses indicated that this initial step was satisfied (Table 6), suggesting that the items represent the same underlying psychological construct in both groups.

**Table 6:** Testing the configural invariance of the measurement tool.

Fit indexes	Good fit	Acceptable fit	Estimations
RMSEA	0 ≤ RMSEA < 0.05	0.05 ≤ RMSEA ≤ 0.10	0.070
NNFI	0.95 ≤ NNFI ≤ 1.00	0.90 ≤ NNFI < 0.95	0.924
CFI	0.95 ≤ CFI ≤ 1.00	0.90 ≤ CFI < 0.95	0.939

CFI, The Comparative Fit Index; NNFI, Non-Normed Fit Index; RMSEA, Root Mean Square Error of Approximation.

In the second stage, metric invariance was assessed by constraining factor loadings to be equal across the clinical and control groups. This level of invariance was supported, as the change in CFI between the configural and metric models was within the acceptable threshold of ±0.01 (Table 7). These findings indicate that the items have comparable meanings across groups.

**Table 7:** Testing the metric invariance of the measurement tool.

Fit indexes	Good fit	Acceptable fit	Estimations
RMSEA	0 ≤ RMSEA < 0.05	0.05 ≤ RMSEA ≤ 0.10	0.068
NNFI	0.95 ≤ NNFI ≤ 1.00	0.90 ≤ NNFI < 0.95	0.928
CFI	0.95 ≤ CFI ≤ 1.00	0.90 ≤ CFI < 0.95	0.940

CFI, The Comparative Fit Index; NNFI, Non-Normed Fit Index; RMSEA, Root Mean Square Error of Approximation.

In the third stage, scalar invariance was tested by constraining item intercepts to be equal across groups. The difference in CFI between the metric and scalar models remained within ±0.01 (Table 8), supporting scalar invariance and suggesting that group comparisons of latent means are appropriate.

**Table 8:** Testing the scalar invariance of the measurement tool.

Fit indexes	Good fit	Acceptable fit	Estimations
RMSEA	0 ≤ RMSEA < 0.05	0.05 ≤ RMSEA ≤ 0.10	0.073
NNFI	0.95 ≤ NNFI ≤ 1.00	0.90 ≤ NNFI < 0.95	0.918
CFI	0.95 ≤ CFI ≤ 1.00	0.90 ≤ CFI < 0.95	0.930

CFI, The Comparative Fit Index; NNFI, Non-Normed Fit Index; RMSEA, Root Mean Square Error of Approximation.

Finally, strict invariance (equality of item residuals) was evaluated by constraining item error variances across groups. However, the change in CFI between the scalar and strict models exceeded the acceptable threshold (ΔCFI = −0.024), indicating that strict invariance was not supported (Table 9). Therefore, subsequent interpretations were based on the established configural, metric, and scalar invariance levels.

**Table 9:** Testing the strict invariance of the multidimensional cognitive attentional syndrome scale.

Fit indexes	Good fit	Acceptable fit	Estimations
RMSEA	0 ≤ RMSEA < 0.05	0.05 ≤ RMSEA ≤ 0.10	0.082
NNFI	0.95 ≤ NNFI ≤ 1.00	0.90 ≤ NNFI < 0.95	0.897
CFI	0.95 ≤ CFI ≤ 1.00	0.90 ≤ CFI < 0.95	0.906

CFI, The Comparative Fit Index; NNFI, Non-Normed Fit Index; RMSEA, Root Mean Square Error of Approximation.

### Convergent validity

Finally, correlation analysis was used to determine the relationships between variables. Convergent validity was assessed by examining the correlations between the MCASS subscales and theoretically related constructs (anxiety, depression, and rumination). No formal correction for multiple testing was applied (e.g., Bonferroni). To further evaluate convergent validity, correlations were examined with instruments based on the same theoretical framework (e.g., CAS-1, MCQ-30) as well as related but theoretically distinct constructs (e.g., experiential avoidance). Correlations between the MCASS subscales and DASS-21, MCQ-30, and RRS-10 were also calculated (Table 10).

**Table 10:** Correlations between multidimensional cognitive attentional syndrome scale, its subscales, and relevant scales’ scores.

Relevant scales	MCASS-RU	MCASS-SU	MCASS-EF	MCASS-TS	MCASS-BA	MCASS-IF	MCASS-W
MEAQ-BA	0.072	−0.141	0.040	0.191^a^	0.256^b^	0.016	0.113
MEAQ-DA	0.290^b^	0.160	0.105	0.125	0.285^b^	0.186^a^	0.413^b^
MEAQ-P	0.163	0.110	0.109	0.201^a^	0.220^a^	0.072	0.172
MEAQ-DS	0.125	0.099	−0.007	0.576^b^	0.316^b^	0.008	−0.021
MEAQ-DS	0.178	0.139	0.107	0.022	0.133	0.147	0.241^b^
MEAQ-DE	−0.180	−0.011	−0.087	0.110	−0.118	0.026	−0.154
MEAQ-T	0.214^a^	0.129	0.082	0.433^b^	0.365^b^	0.150	0.240^b^
DASS-D	0.361^b^	0.181	0.205^a^	−0.190^a^	−0.008	0.232^a^	0.559^b^
DASS-S	0.378^b^	0.197^a^	0.209^a^	−0.178	−0.017	0.251^b^	0.511^b^
DASS-A	0.329^b^	0.175	0.271^b^	−0.120	−0.002	0.264^b^	0.584^b^
RRS-10-B	0.368^b^	0.153	0.140	−0.135	0.054	0.257^b^	0.651^b^
RRS-10-R	0.430^b^	0.199^a^	0.239^a^	0.098	0.215^a^	0.481^b^	0.414^b^
MCQ-PMB	0.232^a^	0.217^a^	0.292^b^	−0.012	0.078	0.234^a^	0.329^b^
MCQ-UCB	0.561^b^	0.209^a^	0.291^b^	0.047	0.119	0.489^b^	0.664^b^
MCQ-CC	0.160	0.081	0.073	0.007	0.234^a^	0.179	0.156
MCQ-NCT	0.469^b^	0.163	0.260^b^	0.067	0.243^b^	0.340^b^	0.643^b^
MCQ-CSC	0.299^b^	0.214^a^	0.257^b^	0.006	0.158	0.423^b^	0.358^b^
CAS1-CAS	0.507^b^	0.369^b^	0.182	0.320^b^	0.267^b^	0.352^b^	0.512^b^
CAS1-MCB	0.480^b^	0.240^b^	0.269^b^	0.122	0.165	0.377^b^	0.522^b^

^a^*p* < 0.050; ^b^*p* < 0.010. A, Anxiety; B, Brooding; BA, Behavioral avoidance; CAS1, Cognitive attentional syndrome; CC, Cognitive confidence; CSC, Cognitive self-consciousness; D, Depression; DA, Distress aversion; DASS, Depression Anxiety Stress Scale; DE, Distress endurance; DS, Distraction and suppression; EF, External Fixation; IF, Internal Fixation; MCASS, Multidimensional Cognitive Attentional Syndrome Scale; MCB, Metacognitive beliefs; MEAQ, Multidimensional Experiential Avoidance Questionnaire; MCQ, Metacognitions Questionnaire; PMB, Positive metacognitive beliefs; NCT, Need to control thoughts; P, Procrastination; R, Reflection; RRS-10, Ruminative Response Scale-10; RU, Rumination; S, Stress; SU, Substance Use; T, Total score; TS, Thought Suppression; UCB, Uncontrollability beliefs; W, Worry.

### Test–retest analyses

Test–retest reliability was examined in a convenience subsample of the control group (*n* = 23) over a 2-week interval. Intraclass correlation coefficients (ICCs) showed moderate to good temporal stability for the MCASS total score and subscales. The ICC for the total score was 0.705 (95% CI: 0.369–0.879). Subscale ICCs were 0.727 for Rumination, 0.717 for Substance Use, 0.741 for External Fixation, 0.736 for Thought Suppression, 0.620 for Behavioral Avoidance, 0.621 for Internal Fixation, and 0.692 for Worry.

## Discussion

The MCASS was originally developed as a 21-item, seven-factor Likert-type measurement tool. In the current study we aimed to report the psychometric features of the Turkish MCASS and to show its usefulness in clinical and non-clinical settings. Several analyses were performed in a patient group and a control group. Measurement invariance analyses were also performed.

The fit and structure of the seven-factor model MCASS-21 was tested using CFA. Root Mean Square Error of Approximation (commonly referred to as the RMSEA), NNFI, CFI, and Standardized Root Mean Square Residual (commonly referred to as the SRMR) fit indices were achieved in both the patient and control groups, supporting the seven-factor structure defined in the original measurement tool was verified for the Turkish culture. The exclusion of Item 7 appears to reflect both linguistic and conceptual issues in the Turkish context. The original item, “I stay alert or watchful at all times,” was translated as “Her zaman uyanık ve dikkatli olurum.” However, this expression may not clearly convey attention directed toward external stimuli, which is the intended meaning of the item within the external fixation dimension of the original scale. Instead, the Turkish wording may be interpreted more generally as a state of alertness or vigilance, without a clear external attentional focus. This semantic ambiguity likely contributed to the weak factor loadings observed in both the clinical and non-clinical samples. Therefore, the validated form reported in the present study should be regarded as a modified 20-item Turkish version of the MCASS rather than a direct item-for-item replication of the original scale. The internal consistency (Table 5) analyses revealed that the reliability values obtained for the subscales of the MCASS varied between 0.694 and 0.954. Reliability values of 0.70 and above indicate good reliability (Salvucci et al., 1997), and values between 0.60 and 0.70 are considered acceptable. It was determined that F3 had acceptable reliability for the patient group. However, the measurements obtained from the other subscales had a good fit. All reliability values from the control group had a good fit. These results are similar to previous reports (Conboy et al., 2021; Helmy et al., 2023).

Convergent validity was tested by correlating MCASS subscale scores and total scores with similar scales (i.e., CAS-1 and MEAQ) as well as the RRS subscales, the MCQ, and the DASS-21 subscales (namely the DASS-A: Anxiety subscale, DASS-D: Depression subscale, and DASS-S: Stress subscale). As expected the MCASS rumination and worry subscale scores moderately correlated with each other. Ruminative responses and MCBs, except for cognitive confidence, were also correlated (Table 10). The MCASS substance use subscale was moderately correlated with the CAS-1 subscale of CAS components but was weaker for other subscales (Table 10). The MCASS external fixation subscale score weakly correlated with some MCBs. The MCASS thought suppression subscale scores were moderately correlated with the MEAQ distraction and suppression subscale score as well as the MEAQ total score and CAS subscale score of CAS-1. The MCASS behavioral avoidance subscale score was moderately correlated with the MEAQ distraction and suppression subscale, whereas the other correlations were weaker. The MCASS internal fixation subscale score was moderately correlated with the ruminative response (reflection) score, uncontrollability metacognitions, cognitive self-consciousness, CAS-1 CAS, and MCB subscales.

Correlations between the DASS subscales (i.e., depression, anxiety, and stress) and the MCASS subscales suggest specific relationships between CAS forms and emotional symptoms (Kowalski and Dragan, 2019). Worry and rumination were highly significantly correlated with CAS forms of depression, anxiety, and stress scores. This is in line with the emphasis on worry and rumination as fundamental elements of the CAS (Papageorgiou, 2006). Internal and external attention have weak correlations between the three subscales of the DASS. The relationships between the symptoms of depression, anxiety, and stress were similar, indicating these symptom groups are highly interrelated (Lee et al., 2019). These results also highlight the transdiagnostic nature of the CAS components.

Test–retest findings indicated moderate to good temporal stability; however, these results should be interpreted cautiously given that the analysis was conducted in a small convenience subsample of controls.

It was determined after the measurement invariance analyses that there were higher fit values in the control group and higher validity in measuring the CAS of healthy individuals because the configural invariance, metric invariance, and scalar invariance was achieved while strict invariance was not. Accordingly, the adapted measurement tool had partial measurement invariance. No study to date has tested the measurement invariance of any metacognitive measurement tools pertaining to the CAS (namely CAS-1 and MCASS). Our results suggest that MCASS may be useful in non-clinical settings.

The results of the multi-group confirmatory factor analysis supported configural, metric, and scalar invariance across clinical and non-clinical groups. It should be noted that strict invariance was not fully achieved in this study. This suggests that while participants in both groups interpret the items in the same way (metric invariance), there may be differences in item-level residual variances. This finding is common in clinical validation studies and does not necessarily undermine the utility of the scale for comparing means across groups. Consequently, the Turkish MCASS can be considered a valid tool for comparing latent factor means between clinical and healthy populations.

Although our results indicated that using the MCASS to evaluate CAS forms, neither the CAS-1 nor the MCASS appear to measure diagnosis-specific CAS components. In the MCT literature several diagnosis-specific metacognitive measurement tools like the Thought Fusion Instrument and the Beliefs About Rituals Inventory, which are used to validate the metacognitive model of obsessive-compulsive disorder (OCD) (Myers et al., 2009; Bose, 2014), strengthen the argument that diagnosis-specific measurement tools are essential to test specific theories. Fergus et al. (2013) evaluated the relationship between specific symptoms and CAS components in a patient sample. They found that specific CAS components had a unique effect on the symptoms of depression, generalized anxiety, and panic/agoraphobia but not on symptoms of social anxiety disorder and OCD.

The existing scales, including the CAS-1 and the MCASS, tend to measure broad and common elements of the CAS related to general anxiety and depression symptoms. For instance, safety-seeking behaviors in social anxiety disorder (McManus et al., 2008) and compulsions in OCD (Steketee and Nishith, 1995) are prominent CAS elements that are not addressed in any scales that intend to measure dysfunctional CAS strategies. We believe this issue may be addressed by measuring the incremental validity of the MCASS in a much larger patient sample. The current results also confirmed that the CAS components are transdiagnostic and are therefore present in various mental disorders. While diagnosis-specific MCT instruments may develop a personalized assessment and monitoring protocols, the MCASS shows promise for transdiagnostic purposes. Although the removal of Item 7 improved the psychometric performance of the Turkish form, this modification may have implications for content coverage within the external fixation domain and should be considered in future cross-cultural research.

## Conclusion

No version of the MCASS has been used to assess and monitor metacognitive strategies until this current study. Our results showed that the Turkish MCASS-20 version is a valid, reliable tool for measuring CAS components in patients and controls. Due to the lower fit scores of the MCASS to measure CAS components in the patient group, its transdiagnostic nature, and the above-mentioned discussions, diagnosis-specific and validated CAS measurement tools are still needed.

## Data Availability

The raw data supporting the conclusions of this article will be made available by the authors, without undue reservation.
