# Supplementary material for: Psychometric properties and measurement invariance of the Turkish version of the multidimensional cognitive attentional syndrome scale
Source: Front Psychol. 2026 May 15;17:1787616. doi: 10.3389/fpsyg.2026.1787616 (PMC13219041; doi:10.3389/fpsyg.2026.1787616)
Supplement: Supplementary file 1 [file Table_1.docx]

|  |  | Kontrol | | Hasta | |  |
| --- | --- | --- | --- | --- | --- | --- |
| Değişkenler |  | f | % | f | % | χ^2^ (p) |
| Cinsiyet | Kadın | 83 | 72.8 | 94 | 81.7 | 2.60  (.107) |
|  | Erkek | 31 | 27.2 | 21 | 18.3 |  |
| Medeni Durumu | Bekar | 94 | 82.5 | 81 | 70.4 | 4.59  (.032) |
|  | Evli | 20 | 17.5 | 34 | 29.6 |  |
| Çalışma durumu | İşsiz | 5 | 4.4 | 30 | 26.1 | 99.58^1^  (.000) |
|  | Öğrenci | 15 | 13.2 | 50 | 43.5 |  |
|  | İşçi | 12 | 10.5 | 19 | 16.5 |  |
|  | Memur | 82 | 71.9 | 12 | 10.4 |  |
|  | Emekli | -- | -- | 2 | 1.7 |  |
|  | Diğer | -- | -- | 2 | 1.7 |  |
| Birlikte yaşadığı kişiler | Yalnız | 37 | 32.5 | 5 | 4.3 | 30.54^1^  (.000) |
|  | Anne/Baba | 35 | 30.7 | 58 | 50.4 |  |
|  | Eş/Partner | 32 | 28.1 | 37 | 32.2 |  |
|  | Arkadaş | 9 | 7.9 | 7 | 6.1 |  |
|  | Çocuklar | 1 | 0.9 | 1 | 0.9 |  |
|  | Kayıp veri | -- | -- | 7 | 6.1 |  |
| Kim tarafından büyütüldünüz? | Yalnız anne | 4 | 3.5 | 12 | 10.4 | 6.48^1^  (.044) |
|  | Anne ve baba | 108 | 94.7 | 96 | 83.5 |  |
|  | Akrabalar | 1 | 0.9 | 3 | 2.6 |  |
|  | Yurt | 1 | 0.9 | -- | -- |  |
|  | Kayıp veri | -- | -- | 4 | 3.5 |  |
| Anne baba ayrı mı? | Var | 4 | 3.5 | 10 | 8.7 | 2.80  (.094) |
|  | Yok | 110 | 96.5 | 103 | 89.6 |  |
|  | Kayıp veri | -- | -- | 2 | 1.7 |  |
| Göç | Var | 8 | 7.0 | 24 | 20.9 | 9.48  (.002) |
|  | Yok | 106 | 93.0 | 89 | 77.4 |  |
|  | Kayıp veri | -- | -- | 2 | 1.7 |  |
| Ebeveynden ayrılık | Var | 6 | 5.3 | 25 | 21.7 | 13.69  (.000) |
|  | Yok | 108 | 94.7 | 88 | 76.5 |  |
|  | Kayıp veri | -- | -- | 2 | 1.7 |  |
| Çocuklukta şiddet görme | Fiziksel | 12 | 10.5 | 25 | 21.7 | 8.34^1^  (.007) |
|  | Cinsel | -- | -- | 3 | 2.6 |  |
|  | Hayır | 102 | 89.5 | 87 | 75.7 |  |
| Geçmişte psikolojik tedavi görme | Yok | 88 | 77.2 | 71 | 61.7 | 29.52^1^  (.000) |
|  | Depresyon | 18 | 15.8 | 6 | 5.2 |  |
|  | YAB | 8 | 7.0 | 27 | 23.5 |  |
|  | PB | -- | -- | 8 | 7.0 |  |
|  | OKB | -- | -- | 2 | 1.7 |  |
|  | SAB | -- | -- | 1 | 0.9 |  |
| Şu an tedavi görme | Yok | 100 | 87.7 | 60 | 52.2 | 71.12^1^  (.000) |
|  | Depresyon | 14 | 12.3 | 6 | 5.2 |  |
|  | YAB | -- | -- | 17 | 14.8 |  |
|  | PB | -- | -- | 28 | 24.3 |  |
|  | SAB | -- | -- | 3 | 2.6 |  |
|  | Kayıp veri | -- | -- | 1 | 0.9 |  |
| İntihar girişimi | Var | 1 | 0.9 | 20 | 17.4 | 19.13  (.000) |
|  | Yok | 113 | 99.1 | 93 | 80.9 |  |
|  | Kayıp veri | -- | -- | 2 | 1.7 |  |
| Sigara içme | Var | 32 | 28.1 | 55 | 47.8 | 9.83  (.002) |
|  | Yok | 82 | 71.9 | 60 | 52.2 |  |
| Alkol alma | Var | 29 | 25.4 | 35 | 30.4 | 0.78  (.377) |
|  | Yok | 85 | 74.6 | 80 | 69.6 |  |
| Madde kullanımı | Var | 2 | 1.8 | 3 | 2.6 | 0.21  (.644) |
|  | Yok | 112 | 98.2 | 112 | 97.4 |  |
| Kronik hastalık var mı? | Var | 18 | 15.8 | 35 | 30.4 | 7.31  (.007) |
|  | Yok | 96 | 84.2 | 78 | 67.9 |  |
|  | Kayıp veri | -- | -- | 2 | 1.7 |  |
| Eğitim düzeyi | Okur yazar değil | -- | -- | 1 | 0.9 | 38.66^1^  (.000) |
|  | Lise | 19 | 16.7 | 62 | 53.9 |  |
|  | Yüksekokul | 10 | 8.8 | 9 | 7.8 |  |
|  | Lisans | 85 | 74.6 | 43 | 37.4 |  |
| Kardeş sayısı | Yok | 18 | 15.8 | 15 | 13.0 | 6.58  (.254) |
|  | 1 | 36 | 31.6 | 41 | 35.7 |  |
|  | 2 | 27 | 23.7 | 17 | 14.8 |  |
|  | 3 | 15 | 13.2 | 18 | 15.7 |  |
|  | 4 | 7 | 6.1 | 10 | 8.7 |  |
|  | 5 ve üstü | 11 | 9.6 | 4 | 3.5 |  |
|  | Kayıp veri | -- | -- | 10 | 8.7 |  |
| **Toplam** |  | 114 | 100.0 | 115 | 100.0 |  |
|  |  | $\bar{X}$ | S | $\bar{X}$ | S | t (p) |
| Yaş |  | 25.36 | 3.79 | 25.73 | 8.48 | 0.42  (.674) |

Tablo 1. Sosyo demografik değişkenlere ait betimsel istatistikler
